# Sociodemographic Factors and Clinical Conditions Associated to Hospitalization in Influenza A (H1N1) 2009 Virus Infected Patients in Spain, 2009–2010

**DOI:** 10.1371/journal.pone.0033139

**Published:** 2012-03-07

**Authors:** Fernando González-Candelas, Jenaro Astray, Jordi Alonso, Ady Castro, Rafael Cantón, Juan Carlos Galán, Olatz Garin, Marc Sáez, Nuria Soldevila, Maretva Baricot, Jesús Castilla, Pere Godoy, Miguel Delgado-Rodríguez, Vicente Martín, José María Mayoral, Tomás Pumarola, José María Quintana, Sonia Tamames, Angela Domínguez, and the CIBERESP Cases and Controls in Pandemic Influenza Working Group

**Affiliations:** 1 Unidad Mixta Genómica y Salud CSISP-Universitat de València, Valencia, Spain; 2 CIBER Epidemiología y Salud Pública, Madrid, Spain; 3 Subdirección de Vigilancia, Comunidad de Madrid, Madrid, Spain; 4 Departament de Ciències Experimentals i de la Salut, Universitat Pompeu Fabra, Barcelona, Spain; 5 Unitat de Recerca en Serveis Sanitaris, Institut Municipal d'Investigació Mèdica-Institut de Recerca de l'Hospital del Mar, Barcelona, Spain; 6 CIBER Enfermedades Respiratorias, Madrid, Spain; 7 Servicio de Microbiología, Hospital Universitario Ramón y Cajal, Madrid, Spain; 8 Departament d'Economia, Universitat de Girona, Girona, Spain; 9 Instituto de Salud Pública de Navarra, Pamplona, Spain; 10 Departament de Salut, Generalitat de Catalunya, Barcelona, Spain; 11 División de Medicina Preventiva y Salud Pública, Universidad de Jaén, Jaén, Spain; 12 Instituto de Biomedicina, Universidad de León, León, Spain; 13 Servicio de Vigilancia de Andalucía, Sevilla, Spain; 14 Red Española de Investigación en Patologías Infecciosas (REIPI), Madrid, Spain; 15 Fundación Vasca de Innovación e Investigación Sanitarias, Sondika, Spain; 16 Dirección General de Salud Pública, Desarrollo e Innovación, Junta de Castilla y León, Valladolid, Spain; 17 Departament de Salut Pública, Universitat de Barcelona, Barcelona, Spain; University of Liverpool, United Kingdom

## Abstract

The emergence and pandemic spread of a new strain of influenza A (H1N1) virus in 2009 resulted in a serious alarm in clinical and public health services all over the world. One distinguishing feature of this new influenza pandemic was the different profile of hospitalized patients compared to those from traditional seasonal influenza infections. Our goal was to analyze sociodemographic and clinical factors associated to hospitalization following infection by influenza A(H1N1) virus. We report the results of a Spanish nationwide study with laboratory confirmed infection by the new pandemic virus in a case-control design based on hospitalized patients. The main risk factors for hospitalization of influenza A (H1N1) 2009 were determined to be obesity (BMI≥40, with an odds-ratio [OR] 14.27), hematological neoplasia (OR 10.71), chronic heart disease, COPD (OR 5.16) and neurological disease, among the clinical conditions, whereas low education level and some ethnic backgrounds (Gypsies and Amerinds) were the sociodemographic variables found associated to hospitalization. The presence of any clinical condition of moderate risk almost triples the risk of hospitalization (OR 2.88) and high risk conditions raise this value markedly (OR 6.43). The risk of hospitalization increased proportionally when for two (OR 2.08) or for three or more (OR 4.86) risk factors were simultaneously present in the same patient. These findings should be considered when a new influenza virus appears in the human population.

## Introduction

The emergence of a new viral strain of influenza A (H1N1) virus in the spring of 2009 represented the first pandemics of the 21st century [1], [2]. The initial data about the infection were alarming, with apparently high death rates in unusual age group, such as infants and children rather than the elderly. This was explained by lack of exposure to a previous H1N1 influenza A virus which was replaced in 1957 by the H2N2 ‘Asian-flu’ strain [3]. Additionally, alarming greater than expected number of serious infections, even with fatal outcomes, were observed among people with no apparent risk for serious infection by influenza virus when compared with the usual profile of seasonal influenza epidemics [4].

Compared to seasonal epidemics, new influenza pandemics have been characterized by increased transmissibility, higher mortality in young age groups, geographic variability, activity peaks out of the cold season and more than one epidemic wave [5]. As a result, health systems world-wide were stressed and, frequently, overwhelmed by the demand at different settings: primary care centers, emergency units, hospital wards and intensive care units.

Spain was the first European country to report a case of pandemic influenza [6] and the rapid adoption of control measures limited the initial wave of the epidemics to 735 cases [7]. Nevertheless, in light of initial reports on underlying clinical conditions leading to severity of infection with the new influenza virus and the need to analyze the effectiveness of the different measures of control adopted by Spanish authorities, a multicenter study was initiated to evaluate these and other factors during the 2009–2010 pandemic wave.

Most previous published reports have analyzed factors leading to extreme severity, usually defined as death or need for admission to emergency care units, of influenza infection [8]–[12] but, to our knowledge, no study has compared hospitalized patients with influenza-infected controls. In this context, and given that pandemic influenza may represent global health risks, we have analyzed which sociodemographic factors and clinical conditions were associated to hospitalization of confirmed influenza A(H1N1) 2009 virus infected patients in Spain during the first eight months of pandemic influenza.

## Materials and Methods

### Study design

A multicenter study utilizing matched case-controls was conducted and included 36 hospitals and primary care centers from seven Spanish regions (Andalusia, Basque Country, Catalonia, Castile and Leon, Madrid, Navarra, and Valencian Community). Cases and controls were recruited between July 2009 and February 2010. Sample size needed to detect a relative risk (OR) of 1.5 and assuming a prevalence of the investigated factors in outpatients of 0.15, a bilateral significance level α = 0.05 and a power of β = 0.80 was 654. using the criteria proposed by Schlesselman [13]. The most conservative assumptions were adopted and the estimated sample size was increased in 10% to account for possible losses. This resulted in a target sample size of 720 for both cases and controls.

### Selection of cases and controls

A case was defined as a patient admitted to hospital for >24 h with RT-PCR confirmed infection by influenza A(H1N1) 2009 virus [14]. Controls were defined as non-hospitalized persons with RT-PCR confirmed infection by the same pandemic virus and were recruited among patients attending primary care centers. Hospitalized cases excluded nosocomial infections (assigned by onset of symptoms 48 h or more after admission to the hospital). Non-hospitalized controls were matched to hospitalized cases by age (±3 years for patients under 18 y and ±5 y for older patients), province of residence and date of admission to the hospital (±10 days).

### Sociodemographic and pre-existing medical variables

For all the subjects included in the study the following demographic and medical variables were obtained: age, sex, ethnic group, education level, tobacco and alcohol use, pregnancy (for women 15–49 y of age), pneumonia in the 2 previous years, chronic obstructive pulmonary disease (COPD), asthma, heart disease, renal insufficiency, diabetes, HIV infection, disabling neurological disease, neoplasia, transplant, morbid obesity (body mass index, BMI>40), treatment with systemic corticosteroids, treatment with inhaled corticosteroids, treatment with antibiotics in the 90 previous days and vaccination against pandemic and seasonal influenza. The medical conditions, retrieved from the patients' medical records, were classified into two groups according to severity [10] (Table 1). The remaining variables were obtained from direct or phone interviews to the patients (or their parents in the case of infants and children).

**Table 1:** Medical conditions considered in this study classified according to severity [10].

Severity	Medical conditions
**High risk**	Solid organ neoplasia
	Hematological neoplasia
	Renal insufficiency requiring hemodialysis
	Transplant
	Asplenia
	Oral corticosteroid therapy, doses >20 mg/day/15 days in the last month
	Immunosuppressive therapy (chemotherapy or others)
	Autoimmune disease
	Nephritic syndrome
	Disabling neurological disease or severe alteration of psychomotor development
	AIDS
**Moderate risk**	Asymptomatic HIV infection
	Diabetes mellitus
	Congestive or hemodynamically unstable congenital cardiomyopathy
	COPD, defined as respiratory symptoms for longer than 3 months
	Asthma
	Chronic liver disease
	Renal insufficiency not requiring hemodialysis
	Hemoglobinopathy or anemia
	Mental disability: Down syndrome, dementia and others
	Neuromuscular disease
	Convulsions
	Long-lasting therapy with acetylsalicylic acid
	Obesity (with MIC score)
	Pregnancy

### Statistical analyses

Bivariate comparisons for sociodemographic and clinical variables were performed between cases and controls by means of Pearson's chi-square, for categorical variables, and Student's t tests, for normally-distributed continuous variables. Crude Odds Ratios (OR) were estimated using the McNemar chi-square test. To estimate the adjusted odds ratio (aOR) a multivariate analysis using conditional logistic regression was performed including those independent variables found to be associated with both the risk factor and the hospitalization in the previous bivariate analyses. In order to detect those variables that could be associated in the multivariate setting but not in the bivariate one, two additional strategies were carried out: full model (i.e. with all candidate variables) and stepwise backward regression [15]. The interactions between age groups (0–18 years, ≥18 years) and the history of vaccination were analyzed by logistic regression. The analyses were performed with SPSS version 18.

### Ethics

All the information collected was treated as confidential, in strict observance of legislation on observational studies and the principles expressed in the Declaration of Helsinki. The study was approved by the Ethics Committee of the hospitals involved. Written informed consent was obtained from all patients or their parents in the case of children (0–17 years).

## Results

A total of 699 hospitalized and 703 non-hospitalized cases of influenza A (H1N1) 2009 were included in the study. Slightly more than half of them (55%) were recruited after November 16, when the vaccination program with the pandemic vaccine was started in Spain.

The most relevant social and demographic variables for both groups are shown in Table 2. Non-Caucasian ethnic groups (Gypsies, Amerinds and Arabian/North-Africans) were considerably more frequently present among hospitalized than among non-hospitalized cases, with crude OR ranging from 2.74 to 8.20. Lower education level was significantly more frequent among hospitalized patients (OR 0.34, 95% confidence interval [CI] 0.26–0.46). More women were present among non-hospitalized than in hospitalized cases, but after adjustment for other variables (Table S1) sex was no longer statistically significant. We encountered some problems in applying the same matching criterion (±3 years) in the eldest age group (>65 years) than in the other groups and, consequently, we had to increase the age interval considered up to 5 years. This and the low number of non-hospitalized cases among this age group explains the significant difference found in the age group distributions, which disappeared in the adjusted multivariate analysis (Table 2).

**Table 2:** Main sociodemographic features of hospitalized and non-hospitalized patients with confirmed infection by influenza A(H1N1) 2009 virus in Spain 2009–2010.

	Hospitalized cases (n = 699)	Non-hospitalized cases (n = 703)	Crude OR (95% CI)	p value	Adjusted OR (95% CI)	p value
**Age average ± SD**	37.82±22.77	35.72±20.77	1.08 (0.90–1.21)	0.07	1.05 (0.91–1.19)	0.07
**Age group**						
0–4	79 (11.3%)	74 (10.5%)	1		1	
5–14	76 (10.9%)	87 (12.4%)	0.45 (0.19–1.11)	0.08	0.25 (0.03–1.81)	0.17
15–24	57 (8.2%)	81 (11.5%)	0.53 (0.18–1.54)	0.24	0.38 (0.06–2.42)	0.31
25–44	203 (29.0%)	242 (34.4%)	1.04 (0.33–3.26)	0.95	0.53 (0.08–3.47)	0.50
45–65	197 (28.2%)	184 (26.2%)	3.10 (0.92–10.42)	0.08	0.91 (0.13–6.22)	0.92
≥65	87 (12.4%)	35 (5.0%)	26.27(5.94–116.20)	0.001	6.73 (0.77–58.81)	0.08
**Women**	356 (50.9%)	399 (56.8%)	0.78 (0.63–0.97)	0.03	0.82 (0.61–1.12)	0.21
**Ethnic group**						
White	602 (87.2%)	645 (93.6%)	1		1	
Gypsy	15 (2.2%)	2 (0.3%)	8.20 (1.86–36.25)	0.006	8.26 (1.03–66.34)	0.04
Amerind	46 (6.7%)	17 (2.5%)	2.90 (1.630–05.17)	<0.001	2.30 (1.16–4.58)	0.02
Arabian or North-African	17 (2.5%)	6 (0.9%)	2.74 (1.08–6.96)	0.03	2.94 (0.86–10.02)	0.08
Other	10 (1.4%)	19 (2.8%)	0.65 (0.28–1.47)	0.30	0.98 (0.32–2.99)	0.98
**Education level**						
Secondary or higher	381 (57.6%)	521 (77.0%)	0.34 (0.26–0.46)	<0.001	0.44 (0.31–0.63)	<0.001

Crude and adjusted odds-ratios, from bivariate and multivariate (logistic regression) analyses respectively, are shown. Only variables used in the adjusted analyses are reported.

OR = Odds ratio; CI = Confidence interval.

Differences between cases and control groups in the frequency of most risk factors and clinical conditions were statistically significant in the bivariate analyses (Table 3). Relevant exceptions were pregnancy and alcoholism, which were not significant, and renal disease, previous smoking habit and transplant, which were almost significant (p = 0.06). The presence of the condition was associated to increased risk of hospitalization in all cases except for pregnancy (OR 0.77, CI 0.41–1.45). However, a more detailed analysis of this factor showed that pregnancy after week 30 was actually associated to an increased risk of hospitalization (aOR 4.17, CI 1.32–13.18). An interaction between age (<18 and ≥18 years) and pandemic influenza vaccination was observed, and therefore these two groups were considered separately. Coverage with pandemic influenza vaccine was very low in both cases and controls and did not allow deriving statistically significant differences between groups. , but the statistical power was only 2% for <18 years and 3.8% for ≥18 years. No statistically significant differences were observed between cases and controls with regards to seasonal influenza vaccination. The statistical power was also low, but higher than for pandemic vaccine (9.6% for <18 years and 59.3% for ≥18 years).

**Table 3:** Main risk factors and clinical conditions of hospitalized and non-hospitalized patients with confirmed infection by influenza A(H1N1) 2009 virus in Spain 2009–2010.

	Hospitalized cases (n = 699)	Non-hospitalized cases (n = 703)	Crude OR (95% CI)	p value	Adjusted OR (95% CI)	p value
**Smoking habits**						
Current smoker	112 (18.0%)	87 (14.8%)	1.44 (1.04–1.99)	0.03	1.06 (0.65–1.74)	0.81
Former smoker	149 (24.0%)	132 (22.4%)	1.32 (0.98–1.78)	0.06	1.12 (0.73–1.72)	0.59
**Alcoholism**	39 (5.7%)	25 (3.9%)	1.56 (0.93–2.64)	0.09	1.36 (0.69–2.69)	0.37
**Pregnancy**	46 (15.4%)	56 (18.2%)	0.77 (0.41–1.45)	0.37	1.12 (0.52–2.44)	0.77
**Pneumonia in the 2 last years**	73 (15.2%)	42 (6.1%)	3.38 (2.05–5.57)	<0.001	2.29 (1.08–4.84)	0.03
**COPD**	68 (9.8%)	12 (1.7%)	6.68 (3.43–12.99)	<0.001	5.16 (1.98–13.45)	0.001
**Asthma**	116 (16.7%)	79 (11.5%)	1.54 (1.13–2.10)	0.007	1.59 (1.12–2.28)	0.01
**Chronic respiratory distress**	53 (7.6%)	6 (0.9%)	10.37 (4.14–25.99)	<0.001	2.97 (1.02–8.70)	0.04
**Hypertension**	132 (19.2%)	71 (10.7%)	2.45 (1.70–3.53)	<0.001	2.52 (1.10–5.79)	0.03
**Chronic heart disease**	62 (9.0%)	14 (2.1%)	6.62 (3.15–13.93)	<0.001	6.10 (1.43–26.09)	0.01
**Congestive cardiomyopathy**	16 (3.4%)	3 (0.5%)	16.0 (2.12–120.65)	0.01	7.31 (0.70–75.81)	0.09
**Renal insufficiency**	29 (4.2%)	15 (2.3%)	1.93 (1.01–3.68)	0.04	1.87 (0.87–4.03)	0.11
**Nephritic syndrome**	11 (1.6%)	5 (0.8%)	1.75 (0.51–5.98)	0.37	0.60 (0.14–2.62)	0.50
**Diabetes**	86 (12.4%)	19 (2.9%)	6.00 (3.33–10.79)	<0.001	3.26 (1.09–9.80)	0.03
**AIDS/HIV infection**	16 (2.3%)	6 (0.9%)	2.42 (0.93–6.33)	0.07	1.31 (0.39–4.37)	0.66
**Disabling neurological disease**	31 (4.5%)	11 (1.7%)	2.80 (1.36–5.76)	0.01	4.00 (1.24–12.99)	0.02
**Solid organ neoplasia**	35 (5.1%)	18 (2.7%)	1.94 (1.06–3.54)	0.03	1.92 (0.99–3.73)	0.06
**Hematological neoplasia**	25 (3.6%)	7 (1.1%)	3.00 (1.27–7.06)	0.01	10.71 (1.95–58.87)	0.01
**Transplant**	31 (4.5%)	17 (2.6%)	1.68 (0.91–3.13)	0.10	1.54 (0.81–2.52)	0.43
**Obesity CMI≥40**	24 (4.8%)	4 (0.7%)	18.0 (2.40–134.8)	0.005	14.27 (1.67–91.7)	0.01
**Previous antibiotic treatment**	176 (25.4%)	74 (11.0%)	2.72 (1.99–3.72)	<0.001	1.84 (1.06–3.20)	0.03
**Systemic corticosteroids**	59 (8.5%)	23 (3.4%)	2.43 (1.46–4.04)	0.001	2.97 (1.01–8.76)	0.04
**Inhaled corticosteroids**	154 (22.2%)	69 (10.2%)	2,49(1,83–3,39)	0.000		
**Pandemic influenza vaccine**						
Children: 0–17 yrs	3 (1.8%)	0 (0.0%)	0.15 (0.01–2.88)	0.12	0.11 (0.03–2.34)	0.14
Adults: ≥18 yrs	9 (1.8%)	4 (0.8%)	1.75 (0.51–5.98)	0.37	1.14 (0.20–6.52)	0.88
**Seasonal influenza vaccine**						
Children: 0–17 yrs	29 (17.5%)	26 (16.5%)	0.97 (0.54–1.74)	0.92	0.82 (0.41–1.64)	0.57
Adults: ≥18 yrs	103 (20.9%)	104 (22.4%)	0.84 (0.59–1.19)	0.33	0.70 (0.46–1.07)	0.1
**Presence of risk factors**						
Moderate risk	314 (53.2%)	255 (41.2%)	3.21 (2.35–4.39)	<0.001	2.88 (1.90–4.35)	<0.001
High risk	148 (25.1%)	64 (10.3%)	6.86 (4.38–10.74)	<0.001	6.43 (3.45–11.98)	<0.001
**Number of risk factors**						
No Risk factors	256(37.2%)	185(28.3%)	1	-	1	-
1 Risk factor	175 (25.4%)	195(28.3%)	1.35(1.04–1.75)	0.022	1.32 (1.00–1.76)	0.046
2 Risk factors	97 (14.1%)	63 (9.2%)	2.32(1.62–3.30)	<0.001	2.08 (1.41–3.07)	<0.001
≥3 Risk factors	160 (23.3%)	44 (6.4%)	5.48(3.79–7.93)	<0.001	4.86(3.21–7.35)	<0.001

Crude and adjusted odds-ratios, from bivariate and multivariate (logistic regression) analyses respectively, are shown. Only variables used in the adjusted analyses are reported.

OR = Odds ratio; CI = Confidence interval.

Using the two additional strategies to detect possible confounders that were present in the multivariate setting but not in the bivariate one, no additional confounders were found (results not shown here but available upon request).

Adjusted multivariate analyses revealed that only a subset of the previous variables were still significantly different between hospitalized and non-hospitalized influenza patients (see Table S1). Among the non-clinical variables (Table 2), the strongest significant effect was found for the education level, with low levels of education associated to hospitalization (aOR 0.44, CI 0.31–0.63). Some ethnic backgrounds retained marginal significance, in particular Gypsies (aOR 8.26, CI 1.03–66.34) and Amerinds (aOR 2.30, CI 1.16–4.58).

Multivariate analyses (Table 3) showed that the variables associated with the highest risk of hospitalization were obesity (aOR 14.27, CI 1.67–91.7), hematological neoplasia (aOR 10.71, CI 1.95–58.87), chronic heart disease (aOR 6.1, CI 1.43–26.09), COPD (aOR 5.16, CI 1.98–13.45), and disabling neurological disease (aOR 4.0, CI 1.24–12.99). Congestive myocardiopathy showed a high associated risk but without statistical significance (aOR 7.31, CI 0.40–75.81), and the remaining clinical variables retained only marginal statistical significance (0.05>p>0.01) and/or represented a relatively low risk of hospitalization (aOR<3).

Finally, we have analyzed the global effect of risk factors. We have used two different approaches. Firstly, we considered the effect associated to the severity of risk factors by considering cases with at least one moderate or severe risk actor (Table 1). The effects were highly significant (p<0.001) for both categories with higher risk for severe factors (aOR 6.43, CI 3.45–11.98) than for moderate ones (aOR 2.88, CI 1.90–4.35). Secondly, we considered the number of risk factors simultaneously present in each patient regardless their severity. In this case, the higher the number of factors the higher the risk of hospitalization, with an aOR = 4.86 (CI 3.21–7.35) for three or more factors (Table 3).

## Discussion

In this study, we have analyzed the factors associated with increased risk of hospitalization among pandemic influenza virus infected patients using a case-control design with participants from a large data set of Spanish hospitals and primary health care centers. The main risk factors for hospitalization of influenza A (H1N1) 2009 were determined to be obesity (BMI≥40), hematological neoplasia, chronic heart disease, COPD and neurological disease, among the clinical conditions, whereas low education level and some ethnic backgrounds (Gypsies and Amerinds) were the sociodemographic variables found associated to hospitalization.

Our approach for analyzing the factors leading to hospitalization by pandemic influenza virus hospitalization has several salient features. First, it is based on a case-control design; second, the control group consisted of non-hospitalized, influenza-infected patients matched by age group, date of hospitalization/symptoms onset and residence, which allowed to minimize hidden effects of confounding factors; and, finally, it included a relatively large sample size (n = 699 hospitalized and 703 non-hospitalized cases).

Case-control studies are among the most common designs in epidemiological studies because of their merits in cost, effort and yield [16]. However, only a few studies in the context of the new pandemic influenza have used this design, mostly in analyses of vaccine effectiveness [17]–[21]. Although a study comparing hospitalized and outpatient cases of confirmed pandemic influenza infection has been published recently [22], this is one of the first studies comparing hospitalized pandemic influenza patients with a control group of pandemic influenza infected patients who evolved more favorably and did not require hospitalization. By using a matched case-control design we have been able to analyze specifically those variables leading to increased risk of hospitalization in pandemic influenza infected patients after removal of other confounding factors.

Most analyses of factors leading to hospitalization due to pandemic influenza are observational studies on prospective or retrospective cohorts [23]–[25]. Some studies have used the general population as the control group [26]–[29] or the comparison has been made to seasonal influenza-infected patients [30]. The choice of control group is a key issue in the validity of case-control studies [16]. We were interested in analyzing which factors influenced hospitalization in people infected by the pandemic virus without the possible confounding effects of susceptibility to infection. In consequence, we have used non-hospitalized patients with the same diagnostic criterion, age, location, and time of infection by the pandemic virus than the matching case. Gilca et al. [22] also used a case-control design with pandemic influenza virus-infected patients to analyze factors associated to risk of hospitalization and outcome severity. Some conclusions from this analysis differ from those obtained in our study (see below), which might be explained by differences in the studied populations but also in methodology because Gilca et al. did not match cases and controls by age and date of hospitalization as in this report.

Ethnicity has appeared associated to severity of infection by pandemic influenza virus in several studies, with indigenous or foreign groups having a larger risk of hospitalization than resident or non-minority groups [31]–[37]. However, there is no evidence for a biological or genetic basis for these differences [35] which have been found also in previous epidemics and its significance, along with that of the education level, may be attributed more to social than biological causes. Access to health services is legally granted to all the residents in Spain but this does not necessarily mean in equal use of these services by all the groups. Immigration and lower education level may be associated with a delay in accessing the physician's consult which, in turn, might aggravate the clinical condition and lead to hospitalization.

The three main factors associated to increased risk of hospitalization among infected patients were morbid obesity, hematological neoplasia, and COPD, all of which had adjusted ORs higher than 5. Two additional factors, chronic heart disease and congestive cardiomyopathy, also had adjusted ORs larger than 5, but their significance was much lower, 0.01 and 0.09, respectively. Other risk factors identified in our analysis after adjustment included diabetes, previous administration of systemic corticosteroids, chronic respiratory distress, hypertension, previous pneumonia, previous antibiotic treatment and asthma.

Morbid obesity (BMI>40) was the most influential factor for hospitalization in our study. The same result was obtained by Morgan et al. [27] in individuals >20 years (n = 161) when compared to the NHANES cohort. Among 2–19 year patients (n = 200), hospitalization was associated with being underweight (BMI< = 5th percentile). Morbid obesity was also found to be associated with hospitalization by pandemic influenza [23], [38]–[40] but Vasoo et al. [41] and Gilca et al. [22] did not find obesity to be a significant factor for hospitalization.

Asthma was found significantly more often in children hospitalized in Canada with pandemic influenza than those admitted to hospital due to seasonal influenza [30] and its incidence among hospitalized patients was higher than in the general population in Australia and New Zealand [31]. Asthma was also found to be associated to hospitalization and severity of pandemic influenza infection in the global pooled analysis by Van Kerkohve et al. [40].

These results largely confirmed previous reports on factors associated to hospitalization in pandemic influenza virus-infected patients. For instance, Jain et al. [23] identified asthma, COPD, diabetes, immunosupression, chronic cardiovascular disease, chronic renal disease, neurocognitive disorder, neuromuscular disorder, pregnancy and seizure disorder as medical conditions representing an increased risk for complications of pandemic influenza infection. The more conditions present in any individual, the higher the risk of complications leading to hospitalization, especially for patients with 18 or more years of age. This was an observational study of US-wide reported cases with 272 patients evaluated from April 1 to June 9, 2009, during the initial pandemic wave. Since no appropriate control group was analyzed, the authors could not provide estimates of relative risk for these or other factors frequently found among hospitalized patients. This problem was overcome by Yu et al. [29] in their large study of hospitalizations in China due to pandemic influenza by using as control the general population and comparing serious (defined as entrance in ICUs or death) and mild (other courses of the disease) infections providing estimate of relative risks for both affected groups. Similar factors to those of Jain et al. [23] were identified as associated to serious progress of infection. An accumulation of factors was also identified by the UK FLU-CIN [42] to confidently differentiate between pandemic influenza and community acquired pneumonia patients admitted to hospitals. In our analysis, we have determined that both the number and the severity of risk factors are positively associated to increased risk of hospitalization. The presence of at least one high risk factor more than doubles the risk of hospitalization compared to moderate factors (aOR 6.43 and 2.88, respectively). Similarly, the more risk factors present in a patient, regardless their severity, the higher the risk of hospitalization (Table 3). Gilca et al. [22] also found that the presence of at least one underlying medical condition increased significantly the risk of hospitalization. The use of an appropriate matched control group of pandemic influenza patients reinforces these conclusions and the need to monitor closely the presence of these factors in influenza infected patients.

Although slightly more than half (55%) of the cases were included in the study after the launching of the vaccination program with the pandemic formulation, the low coverage with the pandemic vaccine, both in hospitalized and non-hospitalized patients, is remarkable and precluded making any meaningful comparison between the two groups because the statistical power to detect differences was very low. Vaccination coverage with the seasonal influenza vaccine was higher than that of the seasonal influenza vaccine. It would have been reasonable for at least those people vaccinated with the seasonal vaccine to have been vaccinated with the pandemic vaccine, because the majority of cases included in this study appeared once the pandemic vaccine was available in Spain [43]. Possible communication failures on the effectiveness of the vaccine and, especially, its safety [44]–[46], may explain this.

Pregnancy was not a significant factor for hospitalization in our study. Several previous reports have held the opposite. For instance, Louie et al. [39] concluded that H1N1 influenza virus could cause severe illness and death in pregnant and postpartum women in California, but no control group or general cohort was analyzed to establish relative risk or significance values for this assertion. Among women who were hospitalized due to pandemic influenza infection in the USA during 2009, pregnancy appeared to be associated to severity of the infection and death [47] but, once again, no control population was analyzed. Nevertheless, the proportions of hospitalized pregnant women varied largely among studies [48]. Our finding that women in the last weeks of pregnancy do have an increased risk of hospitalization suggests that a more detailed analysis of this factor should be undertaken.

This observational study may have some limitations. One possible limitation is that interviewers knew whether interviewees were cases or controls and this could have influenced information gathering. However, the same protocol was followed for both groups and information on vaccination history and clinical variables was collected from medical records recorded before the study began, so it is unlikely that information bias, if any, affected the results. Another possible limitation is the generalization of our conclusions, which are based on a relatively large but still limited sample, to the general population. This is inherent to most case-control studies, because this design severely reduces sample sizes as compared to those based on population analysis. This potential drawback is compensated, in our opinion, by the large number hospitals and primary care centers involved in the study, which provide a wide representation of the Spanish population.

For clinical physicians, and also for public health managers, it is crucial to establish which factors are associated to severity of influenza infection and to an increased need of hospitalization. Differences in disease progression have multifactorial causes and include biological (from both hosts and pathogens), social, and pre-existing clinical conditions. Replacement of the previous H1N1 strain (Brisbane/59/07) by the new 2009 pandemic strain might be accompanied by a change in progression and other clinical effects, which have to be carefully studied. Some studies have compared the relevant features of previous seasonal and the new pandemic. For instance, Carcione et al. [49] found no relevant differences in factors related to infection and hospitalization between seasonal and pandemic influenza in Western Australia during the 2009 influenza season, a period and place in which both H1N1 strains co-circulated. For the Advisory Committee on Immunization Practice of the United States CDC, persons considered to be at high risk of infection by influenza A (H1N1) virus strains differed between seasonal and pandemic viruses in factors such as neurologic or neurodevelopment conditions, long-term aspirin treatment (for persons aged < = 18 years), being of American Indian or Alaska Native ethnicity, and being aged >65 years instead of 50 [4], [50].

In conclusion, our results show that non-Caucasian ethnic groups and people with low educational level have a higher risk of being hospitalized if pandemic influenza virus infection occurs, as also do people presenting three or more medical conditions. These findings may help establishing which groups should receive special attention when a new influenza virus appears in the human population.

## Supporting Information

Table S1 **Variables used for adjustment in multivariate analyses.**
(DOC)
