# Supplementary material for: Sociodemographic Factors and Clinical Conditions Associated to Hospitalization in Influenza A (H1N1) 2009 Virus Infected Patients in Spain, 2009–2010
Source: PLoS One. 2012 Mar 7;7(3):e33139. doi: 10.1371/journal.pone.0033139 (PMC3296770; doi:10.1371/journal.pone.0033139)
Supplement: Table S1 — Variables used for adjustment in multivariate analyses. (DOC) [file pone.0033139.s001.doc]

**SUPPLEMENTARY TABLE 1.** Variables used for adjustment in multivariate analyses

**Age:** Ethnic group, education level, Smoking habits, Alcoholism, COPD, Asthma, Chronic respiratory distress, Hypertension, Chronic heart disease, Renal Insufficiency, Diabetes,

AIDS/HIV infection, Solid organ neoplasia, Hematological neoplasia, Transplant, Systemic corticosteroids, Inhaled corticosteroids

**Age group:** sex, education level, Smoking habits, Alcoholism, COPD, Asthma, Chronic respiratory distress, Hypertension, Chronic heart disease, Renal Insufficiency, Diabetes, Disabling neurological disease, Solid organ neoplasia, Hematological neoplasia, Previous antibiotic treatment, Systemic corticosteroids, Inhaled corticosteroids

**Sex:** Ethnic group, Smoking habits, Alcoholism, COPD, Asthma, Chronic respiratory distress, Hypertension, Chronic heart disease, AIDS/HIV infection, Transplant, Systemic corticosteroids

**Ethnic group:** sex**,** education level, Smoking habits, Alcoholism, Hypertension

**Education level:** Ethnic group, Smoking habits, COPD, Chronic respiratory distress, Hypertension, Chronic heart disease, Diabetes, Disabling neurological disease, Solid organ neoplasia, Previous antibiotic treatment, Systemic corticosteroids, Inhaled corticosteroids

**Smoking habits:** Sex, Ethnic group, education level, Alcoholism, COPD, Asthma, Chronic respiratory distress, Hypertension, Chronic heart disease, Diabetes, AIDS/HIV infection, Obesity, Previous antibiotic treatment

**Alcoholism:** Sexe, Ethnic group, smoking habits, Chronic heart disease, AIDS/HIV infection, Hematological neoplasia, transplant, Previous antibiotic treatment

**Pregnancy:** Ethnic group, education level, Smoking habits, Asthma, Hypertension, Systemic corticosteroids

**Pneumonia in the 2 last years:** education level, Smoking habits, Alcoholism, COPD, Asthma, Chronic respiratory distress, Diabetes, AIDS/HIV infection, Solid organ neoplasia, Previous antibiotic treatment, Systemic corticosteroids

**COPD:** Sex, education level, Smoking habits, Chronic respiratory distress, Hypertension, Chronic heart disease, Diabetes, Solid organ neoplasia, Previous antibiotic treatment, Systemic corticosteroids, Inhaled corticosteroids

**Asthma:** Sex, Smoking habits, Chronic respiratory distress, Renal insufficiency, Solid organ neoplasia, Hematological neoplasia, Previous antibiotic treatment, Systemic corticosteroids

**Chronic respiratory distress:** Sex, education level, Smoking habits, COPD, Asthma, Hypertension, Chronic heart disease, Diabetes, Solid organ neoplasia, Obesity, Previous antibiotic treatment, Systemic corticosteroids, Inhaled corticosteroids

**Hypertension:** Sex, Ethnic group, education level,smoking habits, COPD, Asthma, Chronic respiratory distress, Chronic heart disease, Renal insufficiency, Diabetes, Solid organ neoplasia, Hematological neoplasia, Transplant, Obesity, Previous antibiotic treatment, Systemic corticosteroids, Inhaled corticosteroids

**Chronic heart disease:** Sex, education level,smoking habits, Alcoholism, COPD, Chronic respiratory distress, Hypertension, Renal insufficiency, Diabetes, Solid organ neoplasia, Hematological neoplasia, Transplant, Obesity, Previous antibiotic treatment, Systemic corticosteroids, Inhaled corticosteroids

**Congestive cardiomyopathy:** education level, COPD, Chronic respiratory distress, Hypertension, Chronic heart disease, Renal insufficiency, Diabetes, Transplant, Systemic corticosteroids

**Renal insufficiency:** Sex, education level, COPD, asthma, Hypertension, Chronic heart disease, Diabetes, Disabling neurological disease, Solid organ neoplasia, Transplant, Previous antibiotic treatment, Systemic corticosteroids

**Nephritic syndrome:** smoking habits, Asthma, Hypertension, Renal insufficiency, Diabetes, Disabling neurological disease, Transplant, Systemic corticosteroids

**Diabetes:** education level, smoking habits, COPD, Chronic respiratory distress, Hypertension, Chronic heart disease, Renal insufficiency, Hematological neoplasia, Transplant, Obesity, Previous antibiotic treatment, Systemic corticosteroids

**AIDS/HIV infection:** Sex, smoking habits, Alcoholism, Systemic corticosteroids

**Disabling neurological disease:** Sex, education level, Renal insufficiency, Obesity

**Solid organ neoplasia:** Sex, Ethnic group, education level, smoking habits, COPD, asthma, Chronic respiratory distress, Hypertension, Chronic heart disease, Renal insufficiency, Diabetes, Hematological neoplasia, Transplant, Obesity, Previous antibiotic treatment, Systemic corticosteroids

**Hematological neoplasia:** Sex, asthma, Hypertension, Chronic heart disease, Diabetes, Transplant, Previous antibiotic treatment, Systemic corticosteroids, Inhaled corticosteroids

**Transplant:** Sex, Ethnic group, education level, smoking habits, COPD, asthma, Chronic respiratory distress, Hypertension, Chronic heart disease, Renal insufficiency, Diabetes, Solid organ neoplasia, Hematological neoplasia, Obesity, Previous antibiotic treatment, Systemic corticosteroids

**Obesity CMI ≥ 40:** education level, smoking habits, COPD, Chronic respiratory distress, Hypertension, Chronic heart disease, Diabetes, Disabling neurological disease, Solid organ neoplasia, transplant

**Previous antibiotic treatment:** education level, smoking habits, COPD, asthma, Chronic respiratory distress, Hypertension, Chronic heart disease, Renal insufficiency, Diabetes, Hematological neoplasia, Transplant, Systemic corticosteroids, Inhaled corticosteroids

**Systemic corticosteroids:** education level, COPD, asthma, Chronic respiratory distress, Hypertension, Chronic heart disease, Renal insufficiency, Diabetes, AIDS/HIV infection, Solid organ neoplasia, Hematological neoplasia, Transplant, Previous antibiotic treatment, Inhaled corticosteroids

**Inhaled corticosteroids:** Education level, COPD, Renal insufficiency, hypertension, Diabetes, Neoplasia, Previous antibiotic treatment, Corticos_S1

**Number of risk factors:** Sex, education level

**Pandemic influenza vaccine:**

0-17 years: education level, diabetes, Hematological neoplasia, Previous antibiotic treatment

≥18 years: COPD, asthma, Renal insufficiency, transplant, obesity, Previous antibiotic treatment, Systemic corticosteroids

**Seasonal influenza vaccine:**

0-17 years: Chronic respiratory distress, Inhaled corticosteroids

≥18 years: Ethnic group, education level, COPD, asthma, Chronic respiratory distress, Hypertension, Chronic heart disease, Renal insufficiency, Diabetes, Solid organ neoplasia, Transplant, Systemic corticosteroids

**Pneumococcal 23-valent vaccine in ≥ 65 years:** COPD, Chronic respiratory distress, Chronic heart disease

**Pneumococcal 7-valent vaccine in ≤ 5 years:** Chronic respiratory distress
